# Predicting bottle-feeding practices among mothers of children aged 0–24 months in Somalia: a machine learning analysis of the 2020 Demographic and Health Survey

**DOI:** 10.3389/fnut.2026.1773963

**Published:** 2026-03-10

**Authors:** Abdifatah Ibrahim Mouse, Hodo Abdikarim, Abdisalam Hassan Muse

**Affiliations:** 1 Faculty of Science and Humanities, School of Postgraduate Studies and Research, Amoud University, Hargeisa, Somaliland; 2 Research and Innovation Center, Amoud University, Borama, Somaliland

**Keywords:** bottle-feeding, children, Demographic and Health Survey, machine learning, Somalia

## Abstract

**Background:**

In Somalia, where infectious diseases and malnutrition pose significant threats to child health, suboptimal infant feeding practices like bottle-feeding are a critical public health concern. This study aimed to determine the prevalence and identify key predictors of bottle-feeding among mothers of children aged 0–24 months using advanced machine learning approaches.

**Methodology:**

We analyzed data from the 2020 Somali Demographic and Health Survey (*n* = 5,416). Eight machine learning algorithms were employed to predict bottle-feeding status based on socioeconomic, demographic, and healthcare-related variables. Model performance was evaluated using accuracy, AUC-ROC, precision, recall, and specificity metrics.

**Results:**

The prevalence of bottle-feeding among children aged 0–24 months who fed the bottle milk was 45.72% (95% CI: 44.39–47.05). Key predictors included household wealth (AOR = 0.66 for rich vs. poor), place of delivery (AOR = 0.76 for facility vs. home delivery), and child's sex (AOR = 1.13 for males). The Random Forest model demonstrated superior performance (Accuracy = 73.68%, AUC = 0.802), with geographic region, residence type, and parity emerging as the most important predictive features.

**Conclusion:**

Bottle-feeding is remarkably prevalent in Somalia and strongly associated with poverty, limited healthcare access, and sociocultural factors. This practice contradicts WHO recommendations and exposes infants to substantial health risks in Somalia's challenging environment.

**Recommendations:**

Targeted interventions should focus on high-prevalence regions, integrate breastfeeding support with poverty reduction programs, improve access to health facilities, and address cultural beliefs through community education. Implementation of predictive models could enhance targeted public health efforts to promote optimal infant feeding practices.

## 1 Introduction

Bottle-feeding, a common infant feeding method, is defined as the practice of feeding an infant any liquid, including expressed breast milk or formula, using a bottle equipped with a nipple or teat (1, 2). While bottle-feeding offers convenience, its impact on infant health and breastfeeding practices necessitates careful examination (3). This research aims to predict bottle-feeding practices among mothers of children aged 0–24 months in Somalia, utilizing machine learning techniques applied to data from the 2020 Demographic and Health Survey (DHS). Understanding the factors influencing bottle-feeding in this context is crucial for informing interventions and promoting optimal infant and young child feeding (IYCF) practices (4).

Infant and young child feeding (IYCF) practices are critical determinants of children's nutritional status and, subsequently, their survival, growth, and overall development (5, 6). Appropriate infant feeding has important implications for immediate and future health, particularly in developing countries, where infectious diseases and malnutrition rates are high (5, 7).

Optimal breastfeeding practices, particularly exclusive breastfeeding for the first 6 months of life, are vital for infant health (8). Breast milk provides the ideal nutrition for infants, offering a balanced mix of nutrients, antibodies, and immune factors that protect against infections (9, 10). Furthermore, breastfeeding promotes bonding between mother and child and has long-term health benefits for both (11). Suboptimal feeding practices, including the early introduction of bottle-feeding, can have detrimental health consequences for infants in developing countries (12). Increased risk of diarrheal diseases, respiratory infections, and malnutrition are frequently linked to inappropriate feeding methods (13, 14). Moreover, suboptimal feeding practices contribute to higher rates of infant morbidity and mortality, posing a significant public health challenge (15). Children in developing countries are more vulnerable to infectious diseases and malnutrition, making appropriate infant feeding crucial for their immediate and future health (13, 16).

The World Health Organization (WHO) recommends exclusive breastfeeding for the first 6 months of life, followed by the introduction of nutritionally adequate and safe complementary foods while continuing breastfeeding for up to 2 years or beyond (17). The WHO strongly discourages bottle-feeding due to its potential negative impacts on breastfeeding duration and exclusivity (9, 18).

Bottle-feeding increases the risk of contamination, particularly in settings with poor sanitation, leading to diarrheal diseases and other infections (19). Artificial nipples from bottle-feeding may lead to nipple confusion when infants are exposed to bottle and breastfeeding methods, resulting in the infant refusing to breastfeed (20, 21). Furthermore, bottle-feeding has been associated with an increased risk of overfeeding, potentially contributing to childhood obesity (22).

The WHO emphasizes the importance of promoting and supporting breastfeeding through various strategies, including the Baby-Friendly Hospital Initiative, which aims to create a supportive environment for breastfeeding mothers and their infants (23). These recommendations are rooted in evidence demonstrating the superior health outcomes associated with exclusive and continued breastfeeding, highlighting the need to minimize bottle-feeding and promote optimal breastfeeding practices (24). Bottle-feeding can lead to several negative health outcomes for infants, especially in resource-limited settings (25). Poor hygiene practices associated with bottle use increase the risk of diarrheal diseases and other infections (26, 27).

The prevalence of bottle-feeding varies across developing countries, reflecting diverse cultural practices, socioeconomic factors, and access to healthcare (28). A study in Namibia reported a bottle-feeding prevalence of 35.7% among children under 2 years of age (11). Similarly, a study in Ghana found that 12% of children under 2 years were bottle-fed (12). In Ethiopia, regional variations exist, with a study in the Oromia region reporting a prevalence of 38% (13). In Holeta town, Central Ethiopia, the prevalence of bottle-feeding was found to be 19.6% (4). Across 20 Sub-Saharan African countries, the pooled prevalence of bottle-feeding was 13.74% (29).

Factors contributing to bottle-feeding in developing countries include maternal employment, perceived insufficient breast milk supply, and cultural beliefs (30, 31). Higher levels of maternal education and household wealth have also been associated with increased bottle-feeding rates in some settings (32, 33). A study in Indonesia found a bottle-feeding prevalence of 37.9% among children aged 0–23 months (34). These findings highlight the need for targeted interventions to promote optimal breastfeeding practices and is associated with the reliance on bottle-feeding in developing countries (11). Understanding the specific drivers of bottle-feeding in different contexts is crucial for designing effective strategies to improve infant and young child nutrition (12).

In Somalia, data on bottle-feeding practices is limited, but the country faces significant challenges related to maternal and child health. High rates of poverty, food insecurity, and conflict contribute to poor nutritional outcomes for infants and young children (35). Despite the importance of IYCF practices, there remains a significant gap in the local research specifically addressing bottle-feeding practices in Somalia. Limited data exists on the prevalence, determinants, and health consequences of bottle-feeding among Somali infants. Furthermore, the lack of evidence-based interventions tailored to the Somali context hinders efforts to improve infant nutrition and diminish mortality.

Given the limited data and the critical need to improve IYCF practices in Somalia, this study is justified. By employing machine learning techniques to analyze DHS data, we aim to identify key predictors of bottle-feeding and inform targeted interventions to promote optimal breastfeeding practices and improve infant health outcomes in Somalia.

## 2 Methods and materials

### 2.1 Study area

Somalia is located in the Horn of Africa and covers a total area of 637,657 square kilometers. It consists of diverse landscapes, including plains, plateaus, and mountains. Somalia has the longest coastline in Africa, stretching approximately 3,333 kilometers from the northern tip, known as the Gulf of Aden, to the east and south, bordering the Indian Ocean. It shares borders with Kenya in the southwest, Ethiopia to the west, and Djibouti to the north, with few seasons fluctuations and regular temperatures ranging from 30 °C to 40 °C. Therefore, the study encompassed Somalia as a whole (including Somaliland), including its urban, rural and nomadic areas, as well as all geographic regions (36).

### 2.2 Data source, study design and period

Secondary data based on cross-sectional study design from the children recode (KR file) of the Somali Demographic and Health Survey (SDHS), which was conducted 2020, was employed. The study was used a data set acquired from the Somalia National Bureau of Statistics (SNBS), which was obtained from the published reports of the Somali Demographic and Health Survey 2020 (36). The survey data collection began in February 2018 and was completed in January 2019 for urban and rural areas. This survey, which is the first of its kind in Somalia, was conducted as part of the global DHS project.

### 2.3 Population and sample size

The target population for this analysis included all mothers of children aged 0–24 months in Somalia. The final analytical sample comprised a total of 5,416 mother from the 2020 SDHS who had complete data for all variables of interest included in this study.

### 2.4 Inclusion criteria

All women of reproductive age (15–49 years) who had at least one child between the ages of 0 and 24 months at the time of the survey were included in the analysis.

### 2.5 Study variables

#### 2.5.1 The outcome variable

The outcome variable for this study was the bottle-feeding status of children aged 0–24 months. This was derived from the mother's response to the question of whether the child was given anything to drink from a bottle with a nipple in the 24 h preceding the survey, coded as a binary variable (0 = No, 1 = Yes) in line with WHO guidelines. The explanatory variables included in this study are detailed in Table 1.

**Table 1:** Explanatory variables.

**Level**	**Variable**	**Categories**
Individual-level factors	Maternal age group	15–19 years; 20–34 years; 35–49 years
	Educational status of the mother	No education; primary; secondary; higher
	Maternal employment	Working; not working
	Wealth status	Poor; middle; rich
	Media access	Yes; no
	Child sex	Male; female
	Child age (months)	0–8; 9–11; 12–17; 18–24
	Parity	< 3 Births; 3–4 births; 5 + births
Community-level factors	Region	Awdal; Woqooyi Galbeed; Togdheer; Sool; Sanaag; Bari; Nugaal; Mudug; Galgaduud; Hiraan; Middle Shabelle; Banadir; Bay; Bakool; Gedo; Lower Juba
	Residence type	Rural; Urban; Nomadic
	Place of delivery	Home; Health Facility
	Health facility	No problem; Big problem

### 2.6 Data pre-processing, management, software and statistical packages

Initial data management, including cleaning, outlier detection, and variable labeling, was performed using STATA version 17.0 (37). Subsequent statistical analyses and machine learning modeling were conducted in the R programming environment (Version 4.3.2) (38). Data were imported and transformed using the “haven” and “dplyr” packages, while the ‘caret' framework was utilized to streamline model training, hyperparameter tuning, and cross-validation. Specific machine learning algorithms were implemented using specialized libraries, including “randomForest”, “xgboost”, “lightgbm”, “gbm”, “rpart”, and “ada” for tree-based ensemble methods, and “glmnet”, “e1071”, “class”, and “nnet” for classification models. Finally, model evaluation and feature interpretability were supported by the ‘pROC', “SHAPforxgboost”, and “shapviz” packages, with all visualizations generated via “ggplot2” and “reshape2”.

### 2.7 Data cleaning

Data cleaning was the initial crucial step after data retrieval and involved several actions to ensure data quality. Cases with missing values for the outcome variable were excluded from the analysis (37).

### 2.8 Imbalanced data handling

The Synthetic Minority Over-Sampling Technique (SMOTE) was applied to the training data to prevent biased model performance. The SMOTE can be applied to address the imbalanced data. SMOTE creates new synthetic samples for the minority class. This technique was chosen as it helps improve model accuracy on the minority class without losing valuable information from the majority class (39, 40).

### 2.9 Data splitting

The dataset was randomly split into training and testing sets (41). Eighty percent of (4,333) of the data was used for training the machine learning models, while 20% (1,083) was reserved for testing the model's performance on unseen dat. This ensured an unbiased evaluation of model performance.

### 2.10 Model selection

Eight different supervised machine learning algorithms were considered for predicting bottle-feeding practices. These models included Logistic Regression (LR), Random Forest (RF), K-Nearest Neighbors (KNN), Support Vector Machine (SVM), Gaussian Naïve Bayes (GNB), eXtreme Gradient Boosting (XGBoost), Decision Tree (DT), and Light Gradient Boosting (LGB). The algorithms were chosen based on their suitability for binary classification problems and their ability to handle complex relationships between variables (42). Model selection was based on performance metrics.

### 2.11 Performance evaluation for predictive model and formulas

**Figure 1:**
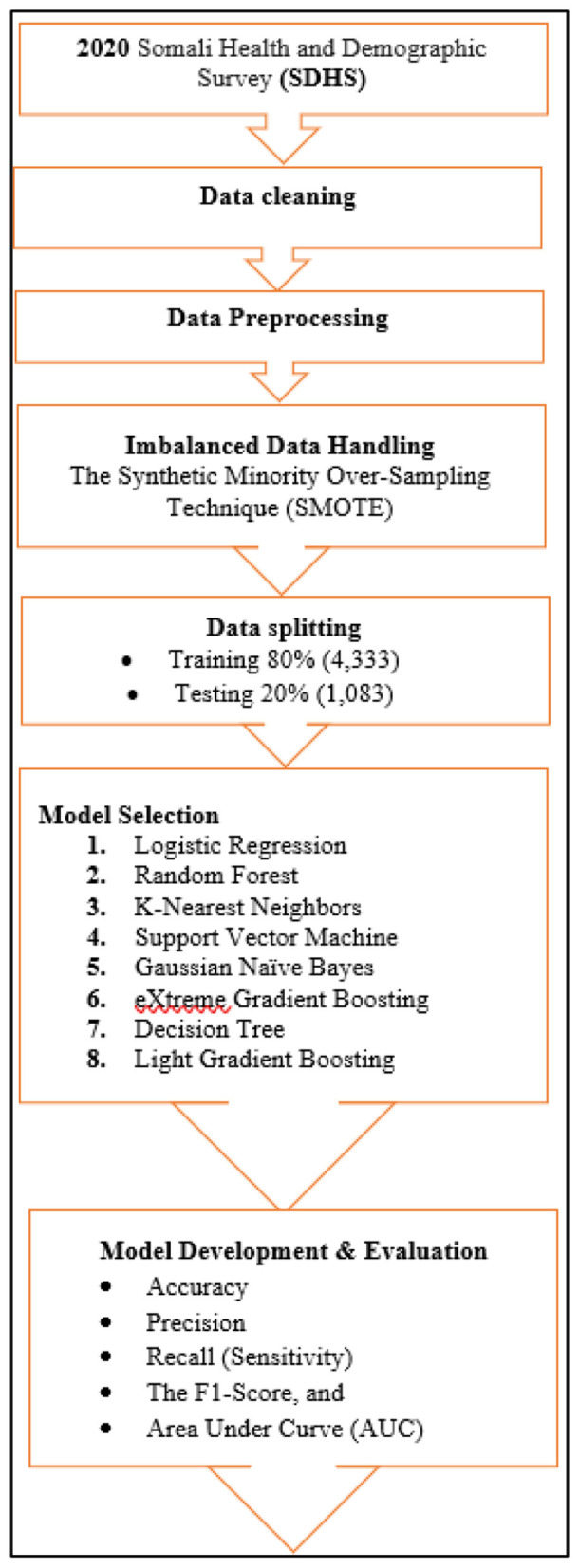
Flow diagram showing machine learning procedure.

The performance of each classifier was rigorously evaluated on the 20% test set using metrics derived from the confusion matrix (43) (Figure 1). The primary metrics included Accuracy, Precision, Recall (Sensitivity), and the F1-Score, which provide a balanced view of model performance. The Area Under the Receiver Operating Characteristic Curve (AUC-ROC) was also calculated to assess the models' ability to discriminate between the two classes (44). These metrics are mathematically defined as:


$$\begin{array}{r} Accuracy=\frac{\left(TP\;+\;TN\right)}{\left(TP\;+\;TN\;+\;FP\;+\;FN\right)}\\ Precision=\;\frac{TP\;}{\left(TP\;+\;FP\right)}\;\\ Recall\;\left(sensitivity\right)=\;\frac{TP\;}{\left(TP\;+\;FN\right)}\\ F1-score=\;\frac{2*\left(Precision*Recall\right)\;}{\left(Precision\;+\;Recall\right)} \end{array}$$

The confusion matrix allowed us to extract one-dimensional performance metrics such as True Positive (TP), True Negative (TN), False Positive (FP), and False Negative (FN) (40).

A comprehensive evaluation using these standard metrics ensures a reliable comparison of the models' predictive capabilities (45).

### 2.12 Ethics approvals

This study involved the secondary analysis of anonymized data from the 2020 Somali Demographic and Health Survey (SDHS). The original survey was implemented by the Somali National Bureau of Statistics (SNBS) and received the necessary ethical clearances (Reference: DDI-SOM-SNBS-SHDS-2020-v1). Since this study utilized a publicly available dataset containing no personally identifiable information, additional ethical approval was not required. Permission to access and use the data was formally granted by The DHS Program (www.dhsprogram.com), and the study was conducted in strict adherence to the data use and confidentiality agreements.

## 3 Results

### 3.1 Prevalence of bottle-feeding in Somalia

The overall prevalence of bottle-feeding with a nipple among the study participants was found to be 45.72% (95% CI: 44.39–47.05) of children under 2 years old in Somalia.

### 3.2 Socio-demographic characteristics of study participants

Table 2 presents the socio-demographic and economic profile of the 5,416 mothers included in the study. The majority of respondents were in the 20–34 age group (73.56%). Educational attainment and workforce participation were notably low; 82.40% of mothers had no formal schooling, and 99.00% reported no employment in the preceding 12 months. Household wealth distribution showed that 45.20% of participants were classified as poor, while 35.43% belonged to the rich wealth quintile. Furthermore, access to information was severely limited, with 96.25% of mothers reporting no exposure to mass media.

**Table 2:** Socio-demographic and background characteristics of the study participants (*n* = 5, 416).

**Variables**	**Categories**	**Frequency**	**Percentage**
Maternal age group	15–19	508	9.38%
	20–34	3,984	73.56%
	35–49	924	17.06%
Maternal education	No education	4,463	82.40%
	Primary	710	13.11%
	Secondary	185	3.42%
	Higher	58	1.07%
Maternal employment	Yes	54	1.00%
	No	5,362	99.00%
Wealth status	Poor	2,448	45.20%
	Middle	1,049	19.37%
	Rich	1,919	35.43%
Media exposure	No	5,213	96.25%
	Yes	203	3.75%
Child's sex	Female	2,573	47.51%
	Male	2,843	52.49%
Parity	< 3 births	1,550	28.62%
	3–4 births	1,599	29.52%
	5 + births	2,267	41.86%
Child's age (months)	0–8	2,019	37.28%
	9–11	460	8.49%
	12–17	1,643	30.34%
	18–24	1,294	23.89%
	Awdal	207	3.82%
	Woqooyi Galbeed	331	6.11%
Region	Togdheer	374	6.91%
	Sool	397	7.33%
	Sanaag	471	8.70%
	Bari	303	5.59%
	Nugaal	323	5.96%
	Mudug	327	6.04%
	Galgaduud	321	5.93%
	Hiraan	267	4.93%
	Middle Shabelle	316	5.83%
	Banadir	655	12.09%
	Bay	134	2.47%
	Bakool	348	6.43%
	Gedo	307	5.67%
	Lower Juba	335	6.19%
Residence	Rural	1,438	26.55%
	Urban	2,277	42.04%
	Nomadic	1,701	31.41%
Health facility access	No problem	2,030	37.48%
	Big problem	3,386	62.52%
Place of Delivery	Home	4,250	78.47%
	Health facility	1,166	21.53%

**Figure 2:**
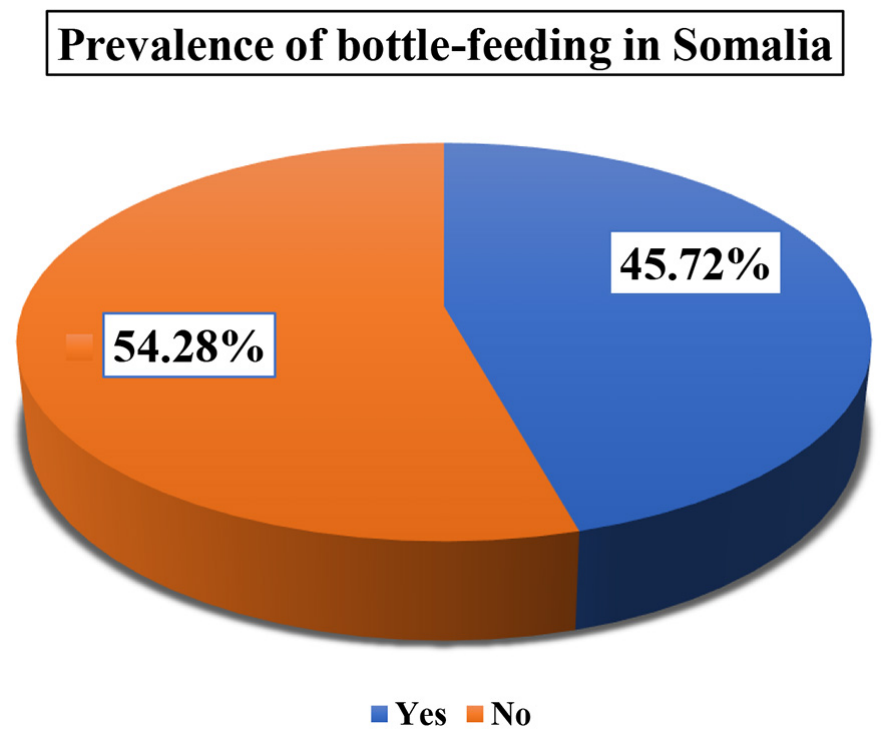
Prevalence of bottle-feeding status in Somalia.

Regarding child and reproductive characteristics, the study population exhibited high fertility, with a plurality of mothers (41.86%) having a parity of five or more births. The sex distribution of the children was relatively balanced, consisting of 52.49% males and 47.51% females (Figure 2). In terms of age, the largest proportion of children were infants aged 0–8 months (37.28%), followed by those aged 12–17 months (30.34%).

Geographically, participants were distributed across all regions, with the highest representation from Banadir (12.09%) and the lowest from Bay (2.47%). Urban residents comprised the largest share of the sample (42.04%), followed by nomadic (31.41%) and rural (26.55%) populations. Healthcare utilization indicators revealed significant barriers; 62.52% of respondents identified distance to health facilities as a “big problem,” and home deliveries were predominant, accounting for 78.47% of births compared to only 21.53% delivered in health facilities.

### 3.3 Bivariate analysis of bottle-feeding practices among

Based on the bivariate analysis in Table 3, several variables demonstrated a statistically significant association with bottle-feeding status, as evidenced by their p-values being less than 0.05. The factors significantly associated with the practice include: Maternal age group (χ^2^ = 16.7824, p = 0.000), maternal education (χ^2^ = 25.8118, p = 0.000), wealth status (χ^2^ = 96.7591, p = 0.000), media access (χ^2^ = 15.2530, p = 0.000), region (χ^2^ = 78.1782, p = 0.000), residence (χ^2^ = 11.1144, p = 0.004), place of delivery (χ^2^ = 64.4228, p = 0.000), and parity (χ^2^ = 17.2547, p = 0.000).

**Table 3:** Bivariate analysis of bottle-feeding status.

**Variables**	**Categories**	**Drank from Bottle**		**Chi-Square**	***p*-value**
		**Yes**	**No**		
Maternal age group	15–19	252 (49.61%)	256 (50.39%)	16.7824	**0.000** ^*^
	20–34	2,135 (53.59%)	1,849 (46.41%)		
	35–49	553 (59.85%)	371 (40.15%)		
Maternal Education	No education	2,492 (55.84%)	1,971 (44.16%)	25.8118	**0.000** ^*^
	Primary	340 (47.89%)	370 (52.11%)		
	Secondary	84 (45.41%)	101 (54.59%)		
	Higher	24 (41.38%)	34 (58.62%)		
Maternal employment	Yes	31 (57.41%)	23 (42.59%)	0.2145	0.643
	No	2,909 (54.25%)	2,453 (45.75%)		
Wealth status	Poor	1,495 (61.07%)	953 (38.93%)	96.7591	**0.000** ^*^
	Middle	559 (53.29%)	490 (46.71%)		
	Rich	886 (46.17%)	1,033 (53.83%)		
Media access	No	2,857 (54.81%)	2,356 (45.19%)	15.2530	**0.000** ^*^
	Yes	83 (40.89%)	120 (59.11%)		
Child sex	Female	1,364 (53.01%)	1,209 (46.99%)	3.1935	0.074
	Male	1,576 (55.43%)	1,267 (44.57%)		
Child's age	0–8 months	45.77%	54.23%	2.07	0.558
	9–11 months	45.65%	54.35%		
	12–17 months	46.87%	53.13%		
	18–24 months	44.20%	55.80%		
Parity	< 3 Births	774 (49.94%)	776 (50.06%)	17.2547	**0.000** ^*^
	3–4 Births	883 (55.22%)	716 (44.78%)		
	5 + Births	1,283 (56.59%)	984 (43.41%)		
Region	Awdal	106 (51.21%)	101 (48.79%)	78.1782	**0.000** ^*^
	Woqooyi Galbeed	164 (49.55%)	167 (50.45%)		
	Togdheer	192 (51.34%)	182 (48.66%)		
	Sool	188 (47.36%)	209 (52.64%)		
	Sanaag	231 (49.04%)	240 (50.96%)		
	Bari	175 (57.76%)	128 (42.24%)		
	Nugaal	200 (61.92%)	123 (38.08%)		
	Mudug	210 (64.22%)	117 (35.78%)		
	Galgaduud	176 (54.83%)	145 (45.17%)		
	Hiraan	166 (62.17%)	101 (37.83%)		
	Middle Shabelle	181 (57.28%)	135 (42.72%)		
	Banadir	318 (48.55%)	337 (51.45%)		
	Bay	68 (50.75%)	66 (49.25%)		
	Bakool	213 (61.21%)	135 (38.79%)		
	Gedo	192 (62.54%)	115 (37.46%)		
	Lower Juba	160 (47.76%)	175 (52.24%)		
Residence	Rural	808 (56.19%)	630 (43.81%)	11.1144	**0.004** ^*^
	Urban	1,265 (55.56%)	1,012 (44.44%)		
	Nomadic	867 (50.97%)	834 (49.03%)		
Health facility access	No problem	1,089 (53.65%)	941 (46.35%)	0.5331	0.465
	Big problem	1,851 (54.67%)	1,535 (45.33%)		
Place of delivery	Home delivery	2,428 (57.13%)	1,822 (42.87%)	64.4228	**0.000** ^*^
	Health facility	512 (43.91%)	654 (56.09%)		

^*^Indicated a statistically significant with *p* < 0.05. Bold values indicate statistical significance at *p* < 0.05.

Conversely, four variables were found to have no statistically significant association with bottle-feeding status in this analysis. These are maternal employment, health facility access and child sex and child's age.

### 3.4 Multivariable logistic regression analysis

In the multivariable logistic regression analysis, after adjusting for potential confounders, wealth status, the sex of the child, place of delivery, and region remained significant predictors of bottle-feeding practices. Interestingly, mothers from middle and rich wealth quintiles had lower odds of bottle-feeding compared to those from poor households (AOR: 0.81, 95% CI: 0.70–0.95 and AOR: 0.66, 95% CI: 0.57–0.76, respectively). Similarly, delivering at a health facility was associated with 24% lower odds of bottle-feeding compared to home deliveries (AOR: 0.76, 95% CI: 0.65–0.88).

Furthermore, the sex of the child showed a significant association, with male children having slightly higher odds of being bottle-fed compared to female children (AOR: 1.13, 95% CI: 1.01–1.26). Regional variations were also evident; for example, mothers residing in the Mudug and Bakool regions had significantly higher odds of bottle-feeding compared to the reference region. Maternal education, age, and media exposure, which were significant in the unadjusted models, lost their statistical significance in the adjusted model (Table 4).

**Table 4:** Adjusted odds ratios for bottle-feeding status.

**Variable**	**Category**	**COR (95% CI)**	***p*-value**	**AOR (95% CI)**	***p*-value**
Wealth status	Poor	1		1	
	Middle	0.73 (0.63, 0.84)	**0.000** ^*^	0.81 (0.70, 0.95)	**0.009** ^*^
	Rich	0.55 (0.48, 0.62)	**0.000** ^*^	0.66 (0.57, 0.76)	**0.000** ^*^
Place of delivery	Home	1		1	
	Health facility	0.59 (0.52, 0.67)	**0.000** ^*^	0.76 (0.65, 0.88)	**0.000** ^*^
Maternal age	15–19	1		1	
	20–34	1.17 (0.98, 1.41)	0.09	1.07 (0.87, 1.32)	0.521
	35–49	1.51 (1.22, 1.88)	**0.000** ^*^	1.26 (0.97, 1.64)	0.08
Region	Awdal	1		1	
	Woqooyi Galbeed	0.94 (0.66, 1.32)	0.708	0.89 (0.62, 1.27)	0.513
	Togdheer	1.01 (0.72, 1.41)	0.976	1.13 (0.80, 1.60)	0.497
	Sool	0.86 (0.61, 1.20)	0.369	1.00 (0.71, 1.41)	0.989
	Sanaag	0.92 (0.66, 1.27)	0.604	0.99 (0.71, 1.38)	0.956
	Bari	1.30 (0.91, 1.86)	0.145	1.16 (0.81, 1.67)	0.411
	Nugaal	1.55 (1.09, 2.21)	**0.015** ^*^	1.40 (0.98, 2.00)	0.067
	Mudug	1.71 (1.20, 2.44)	**0.003** ^*^	1.58 (1.10, 2.27)	**0.013** ^*^
	Galgaduud	1.16 (0.81, 1.64)	0.416	1.05 (0.74, 1.50)	0.782
	Hiraan	1.57 (1.08, 2.26)	**0.017** ^*^	1.43 (0.98, 2.08)	0.062
	Middle Shabelle	1.28 (0.90, 1.82)	0.173	1.29 (0.90, 1.84)	0.168
	Banadir	0.90 (0.66, 1.23)	0.505	0.79 (0.56, 1.10)	0.158
	Bay	0.98 (0.64, 1.52)	0.934	0.90 (0.57, 1.42)	0.649
	Bakool	1.50 (1.06, 2.13)	**0.021** ^*^	1.43 (1.01, 2.04)	0.047
	Gedo	1.59 (1.11, 2.27)	**0.011** ^*^	1.32 (0.91, 1.90)	0.138
	Lower Juba	0.87 (0.62, 1.23)	0.436	0.86 (0.61, 1.23)	0.42
Child's sex	Female	1		1	
	Male	1.10 (0.99, 1.23)	0.074	1.13 (1.01, 1.26)	**0.031** ^*^
Media exposure	No	1		1	
	Yes	0.57 (0.43, 0.76)	**0.000** ^*^	0.79 (0.58, 1.06)	0.115
Residence	Rural	1		1	
	Urban	0.97 (0.85, 1.11)	0.705	1.12 (0.96, 1.30)	0.14
	Nomadic	0.81 (0.70, 0.93)	**0.004** ^*^	0.87 (0.75, 1.01)	0.06

1 shows the reference category.

^*^Indicated a statistically significant with *p* < 0.05. Bold values indicate statistical significance at *p* < 0.05.

### 3.5 Machine learning analysis

**Figure 3:**
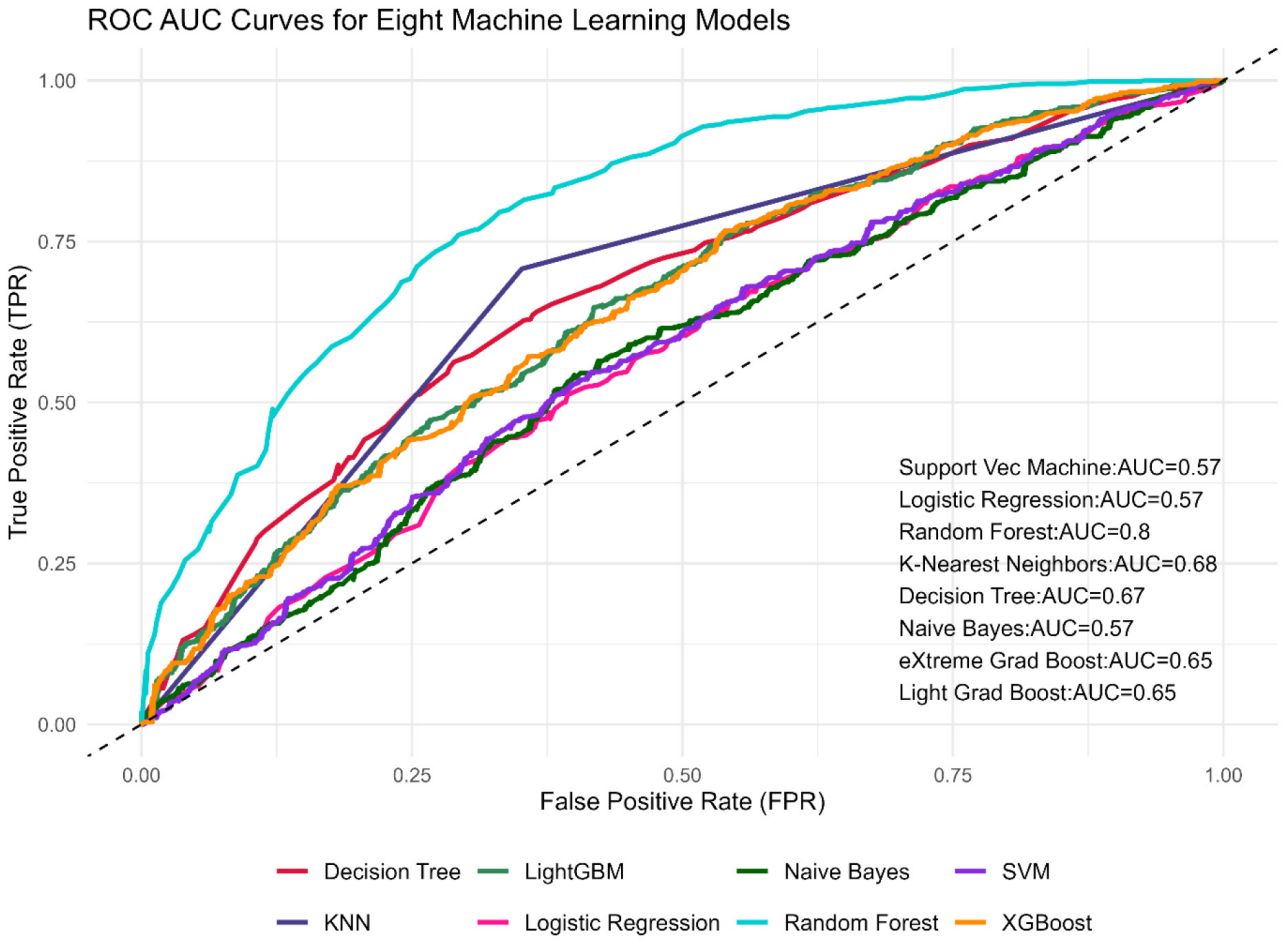
ROC-AUC curve.

Based on the performance metrics in Table 5 and Figure 3, the Random Forest model is the superior algorithm for predicting bottle-feeding practices in this study. It achieved the highest scores across the most comprehensive set of metrics, including the highest Accuracy (73.68%), the highest AUC (0.802), the highest Precision (75.61%), and the highest Specificity (82.99%). Its Sensitivity (62.63%) was also the second highest among all models. The AUC score of 0.802 indicates very good model performance and an 80.2% chance of correctly distinguishing between a child who was bottle-fed and one who was not.

**Table 5:** Confusion matrix.

**MODELS**	**Accuracy**	**F1 Score**	**AUC**	**Sensitivity**	**Precision**	**Specificity**
SVM	0.4414	0.392	0.575	0.3939	0.3900	0.4813
Logistic regression	0.5559	0.503	0.569	0.4909	0.5148	0.6105
Random forest	**0.7368**	**0.685**	**0.802**	**0.6263**	**0.7561**	**0.8299**
KNN	0.6805	0.650	0.678	0.6485	0.6511	0.7075
Decision tree	0.6380	0.610	0.672	0.6202	0.6008	0.6531
Naive bayes	0.5642	0.442	0.569	0.3778	0.5328	0.7211
XGBoost	0.6122	0.532	0.650	0.4828	0.5931	0.7211
LightGBM	0.6113	0.532	0.651	0.4828	0.5916	0.7194

Bold values indicate the highest performance score achieved among the models.

Other models demonstrated varying strengths and weaknesses. The K-Nearest Neighbors (KNN) algorithm recorded the highest Sensitivity (64.85%), meaning it was best at correctly identifying true bottle-fed cases, and also had respectable Accuracy (68.05%) and AUC (0.678). The Decision Tree model also showed moderate performance with an Accuracy of 63.80%. In contrast, the Support Vector Machine (SVM) was the least effective model overall, with the lowest scores in Accuracy (44.14%), AUC (0.575), Sensitivity (39.39%), and Precision (39.00%).

The remaining models Logistic Regression (Accuracy: 55.59%, AUC: 0.569), Naive Bayes (Accuracy: 56.42%, AUC: 0.569), XGBoost (Accuracy: 61.22%, AUC: 0.650), and LightGBM (Accuracy: 61.13%, AUC: 0.651)—all performed below the Random Forest and KNN models. They exhibited low to moderate discriminatory power (AUC ≤ 0.651) and generally lower sensitivity, indicating a weaker ability to correctly identify the positive class (bottle-feeding) compared to the top-performing models.

The Random Forest model exhibited a distinct performance trade-off, prioritizing specificity (82.99%) and precision (75.61%) over sensitivity (62.63%). This performance profile indicates that the model is highly effective at minimizing false positives—thereby reliably confirming actual bottle-feeding cases—though this comes at the cost of a higher false-negative rate, meaning a portion of bottle-feeding mothers may be misclassified as non-bottle-feeding.

### 3.6 Feature selection

The strong performance of the machine learning model, particularly the Random Forest algorithm, is underpinned by a clear and consistent hierarchy of predictive features for bottle-feeding practices. While the exact magnitude of importance varies, the model consistently identifies a core set of dominant predictors. The recurrent appearance of Region, Residence Type, and Parity at the top of the ranking underscores their robustness and fundamental role in determining feeding patterns. This consistency confirms their strong predictive power for this outcome.

**Figure 4:**
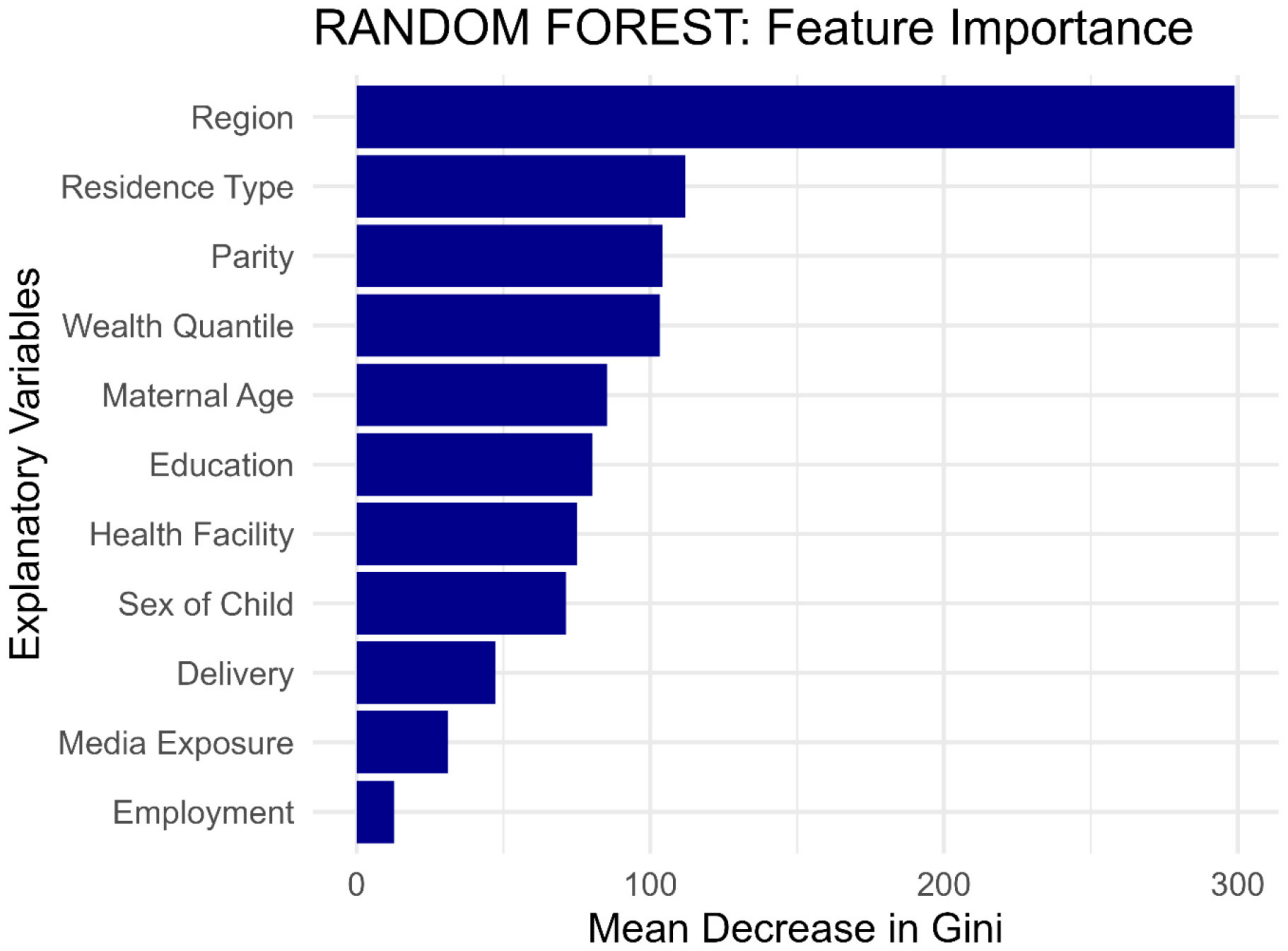
Feature importance.

Furthermore, the model demonstrates a consensus on a secondary tier of influential factors, including Wealth Quantile, Maternal Age, and Education. The agreement across the model's feature selection process highlights the stability and reliability of these findings. It suggests that while geographic and residential context is paramount, socioeconomic status and maternal characteristics are also critical, consistent drivers of bottle-feeding behavior in Somalia. The minimal importance assigned to variables like Employment and Media Exposure across the analysis further reinforces the specific, contextual nature of these predictive relationships (Figure 4).

## 4 Discussion

This study aimed to predict bottle-feeding practices and identify their key determinants among mothers of children aged 0–24 months in Somalia using both traditional statistical and advanced machine learning techniques. The analysis of the 2020 Somali Demographic and Health Survey (SHDS) revealed several critical findings that have significant implications for public health policy and intervention strategies.

The most striking finding is the alarmingly high prevalence of bottle-feeding in Somalia, which stands at 54.28%. This figure is substantially higher than the pooled prevalence of 13.74% reported across 20 Sub-Saharan African countries (29), and rates found in neighboring countries like Ethiopia (19.6%−38%) (4, 13) and Ghana (12%) (12). This indicates that bottle-feeding is not a minor issue but a predominant, normative practice in Somalia, representing a severe public health crisis. In a country grappling with high rates of poverty, food insecurity, and poor sanitation (35), this widespread use of bottles drastically increases the risk of diarrheal diseases, respiratory infections, and malnutrition due to the high potential for contamination (19, 26, 27). This finding directly contravenes WHO guidelines that strongly discourage bottle-feeding due to its negative associations on breastfeeding duration, exclusivity, and infant health outcomes (9, 17, 18).

The multivariable logistic regression analysis identified key independent socio-economic and healthcare determinants of this practice. The strong inverse relationship between household wealth and bottle-feeding is a pivotal finding. Children from rich households had 34% lower odds (AOR: 0.66) of being bottle-fed compared to those from poor households. This challenges the patterns observed in some other developing contexts, where higher wealth is sometimes associated with increased bottle-feeding (32, 33), and instead suggests that in Somalia's fragile state, poverty may force mothers to rely on unsustainable or unsafe substitutes for breastfeeding, or be linked to other unmeasured factors like maternal stress or food insecurity. Furthermore, delivery at a health facility was a significant protective factor, associated with a 24% reduction (AOR: 0.76) in the odds of bottle-feeding. This underscores the critical role of the healthcare system as a primary channel for providing counseling on optimal Infant and Young Child Feeding (IYCF) practices and reinforces the importance of initiatives like the WHO's Baby-Friendly Hospital Initiative (23). The significantly higher odds of bottle-feeding for male children (AOR: 1.13) suggest underlying cultural or son-preference dynamics that merit further qualitative investigation. The strong regional variation, particularly the 58% higher odds in the Mudug region, highlights that localized cultural norms, conflict, access to markets selling formula, or environmental factors are powerful drivers that necessitate targeted, sub-national interventions.

The emergence of geographic region as a dominant predictor highlights deep-seated structural and cultural disparities across Somalia. The elevated prevalence of bottle-feeding observed in regions such as Mudug and Nugaal may be attributed to the predominance of pastoralist lifestyles in these areas, where the cultural practice of introducing animal milk early and the logistical challenges associated with nomadic movement can discourage exclusive breastfeeding. Furthermore, regional variations likely reflect unequal market access; areas situated along major trade corridors may experience a higher availability of imported formula and powdered milk substitutes. Finally, the fragmented nature of Somalia's health infrastructure suggests that access to breastfeeding counseling and humanitarian nutrition support programs varies significantly by state, leaving mothers in underserved regions with less professional guidance on optimal feeding practices.

The application of machine learning models confirmed and refined these insights. The Random Forest (RF) algorithm emerged as the most effective predictive tool, achieving superior performance (Accuracy: 73.68%, AUC: 0.802). Its high precision (75.61%) and specificity (82.99%) indicate it is exceptionally reliable at correctly identifying both bottle-feeding cases and non-cases, making it a valuable tool for targeting interventions. The feature importance analysis from the ML model provided a robust hierarchy of predictors, reinforcing the findings from the traditional analysis while adding a layer of analytical validation. The consensus across models on the paramount importance of Region, Residence Type (urban, rural, nomadic), and Parity confirms that geographical context and maternal birth history are the most fundamental drivers. The agreement on the secondary importance of Wealth, Maternal Age, and Education highlights the complex interplay of socioeconomic and demographic factors. The minimal importance of Maternal Employment and Media Access aligns with the regression results and is likely a reflection of the extremely low prevalence of both in our sample (1% and 3.75%, respectively).

While public health screening tools traditionally prioritize high sensitivity to capture all potential cases, the Random Forest model's trade-off favoring specificity (82.99%) and precision (75.61%) offers strategic value in this specific context. In resource-constrained settings like Somalia, where funding for nutritional interventions is limited, high precision ensures that resources are not wasted on ‘false alarms' (mothers who are not actually bottle-feeding). Consequently, the model serves as a cost-effective targeting tool, identifying a high-confidence cohort for immediate intervention, even though the moderate sensitivity (62.63%) suggests that supplementary broad-based educational campaigns remain necessary to reach the cases missed by the predictive screening.

A critical performance comparison highlights the necessity of employing advanced machine learning algorithms over traditional statistical methods for this specific public health challenge. The Random Forest model achieved an AUC of 0.802, significantly outperforming the Logistic Regression model, which yielded an AUC of only 0.569. This substantial performance gap (AUC = 0.233) indicates that the drivers of bottle-feeding in Somalia involve complex, non-linear interactions—likely between geographic context, parity, and wealth—those standard linear models fail to capture. From a programmatic perspective, the added complexity of the Random Forest model is fully justified; while the Logistic Regression model offers discriminatory power nearly equivalent to random guessing, the Random Forest model provides a robust mechanism for risk stratification. This superior predictive capability renders the machine learning approach far more relevant for policymakers seeking to efficiently target limited intervention resources toward the highest-risk mothers.

## 5 Limitations

This study is subject to several limitations. First, the cross-sectional nature of the DHS data precludes the inference of causal relationships between the identified factors and bottle-feeding practices. Second, the outcome variable relied on a 24-h recall period, which may introduce recall bias and fail to fully capture habitual feeding patterns. Third, critical potential predictors—including maternal knowledge, breastfeeding attitudes, perceived milk insufficiency (30, 31), and the influence of commercial milk formula marketing (3), were not available in the dataset and warrant exploration in future research. Finally, regarding the machine learning analysis, we acknowledge that the Random Forest Gini importance metric can be biased toward high-cardinality variables such as ‘Region.' However, the consistent significance of this variable in the multivariable logistic regression analysis suggests its predictive prominence is a robust finding rather than solely an algorithmic artifact.

## 6 Conclusion

In conclusion, this study employed an integrated analytical approach, combining traditional epidemiology with machine learning, to investigate bottle-feeding practices in Somalia. The analysis revealed that 45.72% of children aged 0–24 months were bottle-fed. Although this represents a lower proportion compared to non-bottle-feeding practices, positioning it as a predominant—rather than marginal—public health challenge. The findings illuminate a complex web of determinants, with geographic, socioeconomic, and healthcare-access factors acting as primary drivers. The superior performance of the Random Forest model (AUC: 0.802) not only confirms the robustness of these predictors but also underscores the value of machine learning in public health for precise risk prediction and intervention targeting. This research fills a critical evidence gap on IYCF practices in Somalia and provides an actionable foundation for reversing trends that contravene global health guidance and endanger child health.

## 7 Policy and programmatic implications

The findings of this study translate into several key policy and programmatic recommendations aimed at mitigating the high prevalence of bottle-feeding and promoting optimal breastfeeding practices in Somalia. Resources and interventions should be prioritized in high-risk regions, such as Mudug, and tailored to specific community types, including nomadic populations. Health messaging should be culturally adapted and delivered through community health workers and local leaders to effectively discourage bottle use and promote breastfeeding.

Given the strong link between poverty and bottle-feeding, nutrition programs should be integrated with broader social protection and economic empowerment initiatives. This could include conditional cash transfers or livelihood support for vulnerable mothers, coupled with counseling on affordable and safe infant feeding options. Such integrated approaches address the underlying economic drivers of inappropriate feeding practices while providing immediate nutritional guidance.

Policy efforts must also focus on increasing access to and use of health facilities for delivery. This involves expanding infrastructure, training healthcare providers on WHO breastfeeding guidelines and lactation support, and fully implementing the Baby-Friendly Hospital Initiative (BFHI) to ensure that health facilities serve as protective environments that promote and support breastfeeding. Facility-based deliveries represent a crucial contact point for educating mothers about the dangers of bottle-feeding.

The success of the machine learning model demonstrates the potential for developing predictive tools to identify high-risk mothers and communities before childbirth. Health management information systems (HMIS) should incorporate such data-driven insights to enable proactive, targeted outreach and resource allocation, moving from a reactive to a preventive public health approach.

Finally, policymakers and funding agencies should support further research to understand the cultural beliefs behind practices like gender-based feeding disparities and the specific barriers faced by mothers in different regions. This deeper understanding is essential for designing context-specific and effective behavior change communication strategies. By implementing these evidence-based policies, Somalia can effectively address the multifactorial drivers of bottle-feeding, align national practices with WHO recommendations, and make substantial progress toward reducing infant morbidity and mortality.

## Data Availability

The raw data supporting the conclusions of this article will be made available by the authors, without undue reservation.
